# A Limb Hypothermia Wearable for Chemotherapy-Induced Peripheral Neuropathy: A Mixed-Methods Approach in Medical Product Development

**DOI:** 10.3389/fdgth.2020.573234

**Published:** 2020-12-15

**Authors:** Jonathan Binder, Ertu Unver, Jane Clayton, Patrick Burke, Richard Paxman, Raghav Sundar, Aishwarya Bandla

**Affiliations:** ^1^Department of Architecture and 3D Design, University of Huddersfield, Huddersfield, United Kingdom; ^2^Paxman Coolers Ltd., Huddersfield, United Kingdom; ^3^National University Cancer Institute, Singapore, Singapore; ^4^Department of Hematology-Oncology, National University Health System, Singapore, Singapore; ^5^Yong Loo Lin School of Medicine, National University of Singapore, Singapore, Singapore; ^6^The N.1 Institute for Health, National University of Singapore, Singapore, Singapore

**Keywords:** evidence-based & research methodology, user-centered accessibility evaluation, limb hypothermia, cryotherapy, supportive care in cancer, chemotherapy-induced peripheral neuropathy (CIPN), medical device design and manufacturing

## Abstract

Chemotherapy-Induced Peripheral Neuropathy (CIPN) is a common dose-limiting side-effect of taxane-based chemotherapy, causing progressive and often irreversible pain/sensitivity in the hands and feet. Prevention/treatments for CIPN are not well-developed and urgently needed. Limb cryocompression during chemotherapy has demonstrated promising early data of preventing/reducing CIPN severity. Currently there are no medical devices available that are dedicated to the specific requirements of CIPN prevention. As part of our ongoing development of a dedicated CIPN-prevention limb cryocompression system, this study documents the design & development of the wearable arm wrap, a central component of the system, from initial concept to a trial-ready prototype. A collaborative and multidisciplinary approach was adopted to address the complex and high-risk nature of this SME (Small Medium Enterprise)-centered medical device design & development process. The complementary collaboration unites multidisciplinary expertise spanning the scope of the project. Alongside the clinical, academic, and design & development expertise, the integration of commercial expertise is imperative to promote the market viability, and ultimate success, of the development. As the global leading experts in scalp cooling specializing in the prevention of chemotherapy-induced alopecia, UK-based SME Paxman Coolers Ltd is optimally positioned to support the commercial and regulatory dimensions. Development and adoption of a novel mixed-methodology (HudPAX) facilitated the integration of evidence-based and user-centered techniques to optimize the design & development approach and ensure integration of all critical design inputs. Alpha prototypes were designed through evidence-based approaches, with data from existing clinical trials utilized to determine the preliminary design inputs, alongside 3D ergonomic data. Investigations utilized computer-aided design, rapid prototyping, additive manufacturing, sketch modeling, and fast ideation. User-based approaches facilitated stakeholder-feedback through expert focus groups, informing further design & development and projecting the design into the next stage, Beta prototyping, for use in large-scale efficacy trials and upscaling manufacturing. This paper demonstrates a novel mixed-methods approach, which promotes cross-sector multidisciplinary collaboration, to address the complex multi-layered challenges posed by an early-stage medical device design & development process.

## Introduction

Chemotherapy-induced peripheral neuropathy (CIPN) is a common debilitating and dose-limiting non-hematological toxicity of taxane-based chemotherapy. Symptoms of CIPN include paresthesias, dysesthesia and pain in the hands and feet, which can be persistent and irreversible even several years after completion of chemotherapy (1–3). A meta-analysis of more than 4,000 chemotherapy-treated patients found the prevalence of CIPN to be 68.1% within the 1st month of chemotherapy treatment, 60.0% at 3 months and 30.0% at 6 months (4). Overall, CIPN significantly increases healthcare costs, and causes long-term morbidity and poor quality of life in cancer survivors (5).

At present, dose modification of the chemotherapy itself remains the most successful approach for the management of CIPN, and symptomatic pharmacological treatment is limited to pain killers. Prevention/treatment for CIPN are not well-developed and urgently needed. Several potential neuroprotective agents have been tested in clinical trials but none have proven effective (6). Recently, there has been a growing evidence base supporting non-pharmacological therapies for mitigating CIPN, especially cryotherapy or hypothermia (6–9).

**Figure 1:**
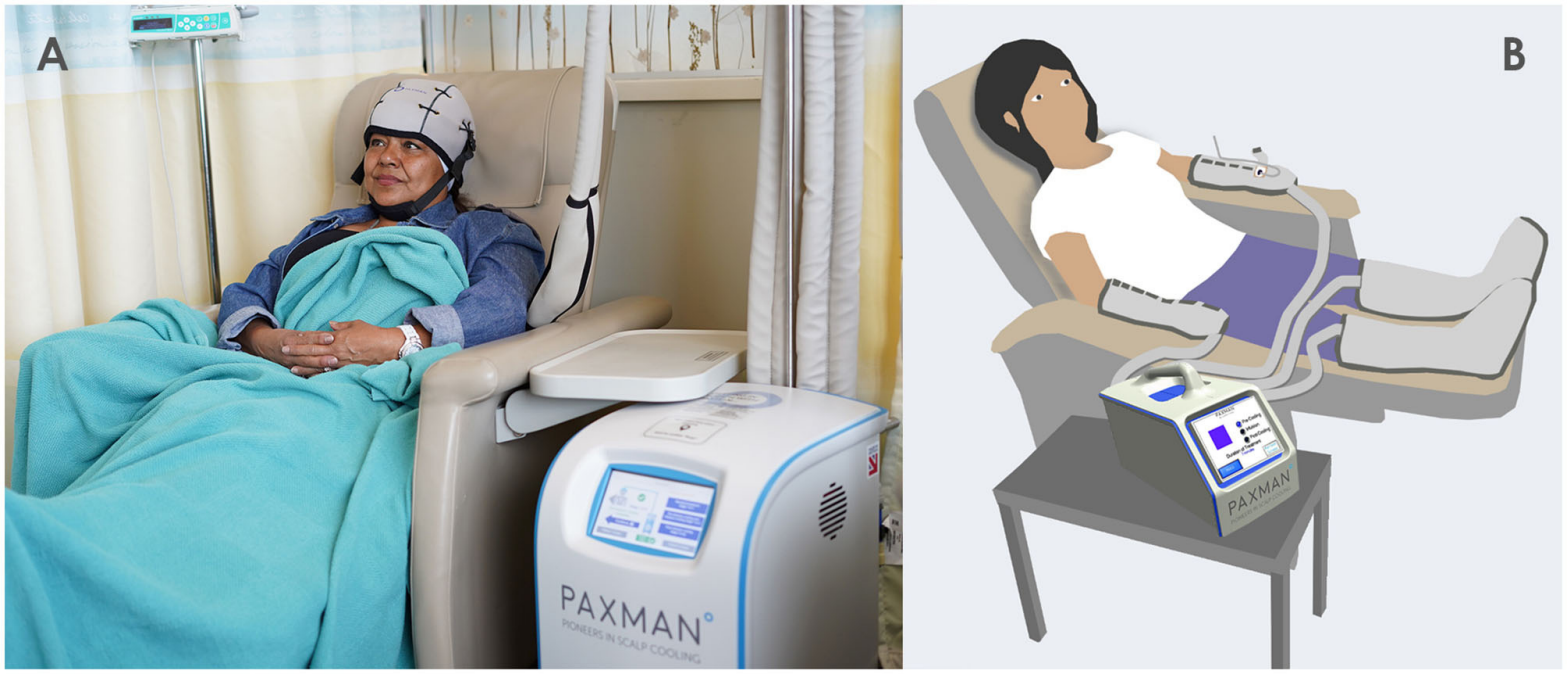
Paxman Scalp Cooling System **(A)**, proposed limb cryocompression system & cooling wraps **(B)**.

Inspired by scalp cooling for preventing chemotherapy-induced alopecia (CIA) (Figure 1A), limb cryotherapy has been studied worldwide and has demonstrated potential for preventing/reducing CIPN severity (10–12). Functioning on the same biological principle as scalp cooling, regional limb cryotherapy induces vasoconstriction and limits the delivery of the toxic chemotherapeutic to the cooled region, and in turn appears to decrease chemotherapy-induced damage. In a recent trial, cryotherapy in the form of frozen gloves appeared to be effective in preventing and reducing the occurrence of CIPN (13). However, the frozen gloves used in this trial have been recalled due to incidences of frostbite and other patient safety issues (14). Moreover, the effect of limb-cryotherapy products utilizing ice/gel packs and frozen gloves is limited due to steep-cooling gradients, non-uniform cooling and lack of thermoregulation causing patient discomfort. In addition, there is a need to frequently replace the frozen gloves or ice packs, causing “breaks” in the cryotherapy delivery (15, 16).

Limb cryocompression during chemotherapy, has demonstrated potential for decreasing the incidence of CIPN (17–19). Cryocompression combines continuous-flow limb cooling and cyclic compression. This modality has demonstrated skin temperature cooling similar to the frozen gloves, but with enhanced levels of patient safety and tolerance. Early feasibility studies in cancer patients were indicative of efficacy, seen as motor nerve protection (19). These studies utilized existing class-I devices that aren't designed specifically for preventing CIPN and include generic cooling devices designed for indications such as sports injury and orthopedic surgical recovery. There are currently no devices which cater specifically to deliver cryocompression for cancer patients for preventing CIPN. Moreover, none of these devices have limb cooling wraps which are suitable for application during chemotherapy infusion.

Due to the emerging scientific and efficacy evidence, there is an imminent demand for a limb cryocompression device, designed specifically for use during chemotherapy infusion for the prevention of CIPN, and this is currently in development. The pilot studies by Bandla et al. facilitated data gathering of user requirements and design inputs for development of a CIPN-specific cryocompression device to facilitate enhanced treatment efficacy (19). The device will integrate a continuous-flow cooling system with multiple disconnectable wearable limb wraps, which deliver compression and cooling therapy to the arms and legs (Figure 1B) with inbuilt sensors to continually monitor and optimize the therapy. The limb cooling wraps are a vital component of this system and address two primary functions, with an internal coolant-containing bladder delivering optimized cooling and compression; and an external insulatory wrap compatible for use during chemotherapy infusion, with optimized comfort and adjustable fit. The design process of the arm wraps is the main focus of this study.

It is acknowledged that the design and development of medical devices is a complex, multilayered and high-risk process, with pitfalls including stringent regulation and commercial challenges. Between 80–90% of new product launches fail and the complex challenges specific to medical device development are likely to increase the failure rate further (20). Small Medium Enterprise's (SME's) in particular face unique challenges implementing product development, due to limited resources & infrastructure, and lack of formalized development processes (20, 21). A collaborative and multidisciplinary approach, uniting all necessary expertise, significantly enhances the prospect of success. The range of challenges arising in medical device design and development, summarized by Grant, include design and development, technological, regulatory, manufacturing, point-of-provision, usage, support, liability, disposal, and business-related (22). In our study, these challenges are comprehensively met by the team's multidisciplinary scope of expertise. Paxman Coolers Ltd, a UK-based SME, are the global-leading experts in scalp cooling, for prevention of CIA, with extensive regulatory, medical-cooling, commercial and market expertise. The University of Huddersfield's product design team have medical-design expertise, with a focus specifically on medical cooling devices. The National University of Singapore, together with the National University Hospital, Singapore, complement this collaboration with their expertise on limb cryocompression in the prevention of CIPN, having established proof-of-concept of safety, tolerability and efficacy in phase-I clinical trials. The power of collaboration between academia and industry for medical device development, has been demonstrated by the University of Huddersfield through the highly successful design & development of the Paxman Scalp Cooling cap (23).

Selection of the optimal design methodology ensures progress through the complex, collaborative and stringent process of medical device design and development. An optimized methodology promotes collaboration across different disciplines and perspectives, and ensures that user needs are comprehensively identified through means suited to the context of the product being designed. An extensive range of design and development methodologies exist that companies can adopt during product development processes, however there is no standard method, and guidance is lacking on the selection and application of the appropriate methodologies for medical device design and development (24). Due to the complexity of this medical device design project, a mixed-methods approach named HudPAX has been developed and applied in this study.

This study documents the design & development of an arm cryocompression wrap from a concept to a trial-ready prototype using the HudPAX approach. Prototype development is essential to managing individual expectations within a project, aiding tangible feedback and justifying the cost and time invested in their development. This study explores the Alpha prototyping stage. The Alpha prototype is the initial attempt at designing and fabricating the product to meet the Product Requirements Specification; the Beta prototype development follows on from this and incorporates the design refinements found in Alpha development and implements them into production. This study generated an Alpha prototype of CIPN-prevention cryocompression arm wraps as a medical device in the feasibility, pre-clinical stage, addressing the complex challenges posed, through a mixed-methods approach.

## Methods

**Figure 2:**
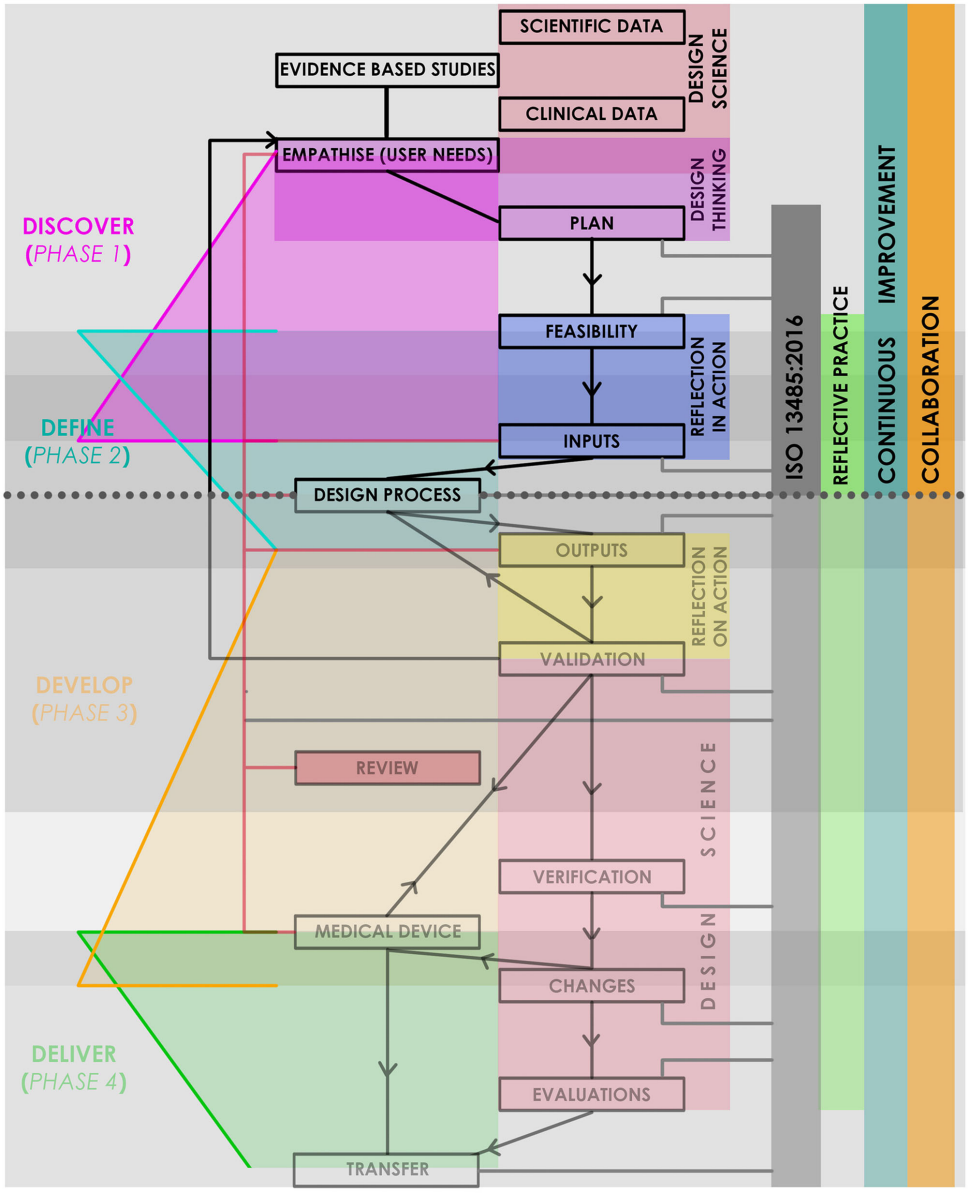
HudPAX Medical Device Development Hybrid method.

The research method designed and applied in this study supports the delicate balance within the project, between the distinct needs of medical device design, commercial feasibility and the stringent regulatory control. In addition, it ensures a comprehensive range of design inputs (user requirements) are successfully gathered and addressed through design outputs. This approach, named HudPAX (Figure 2), integrates the following established design and development research methods:

- The Double Diamond process, by the Design Council: a strategic and incremental process encompassing four stages; Discovery, Define, Develop and Deliver (25);
- Reflective Practice, by Donald Schon: Promoting the reflection on our actions in design so as to engage in a process of continuous learning (26);
- Design Science: an evidence/outcome-based methodology used to bridge the practice–academia divide by developing actionable knowledge that is grounded in evidence (27);
- Design Thinking: This user-based approach is a procedure for investigating potential product-users wants and needs, that culminates in a design brief identifying an opportunity for developing a new product (28).

Integration of the design principles of the Double Diamond process with Design Science (evidence-based approach) and Design Thinking (user-centered approach), through the mixed-methods HudPAX approach, promoted the establishment of an iterative design process which successfully led to the selection of an initial concept for pre-clinical prototyping.

The HudPAX method was developed in-line with the scope of medical regulatory standards, to ensure resulting medical devices are fully compliant with international regulations and to ease the eventual regulatory approval processes. ISO 13485:2016 is the Regulatory Quality management system for medical devices, and the incremental milestones defined by ISO 13485:2016 act as a core element within the HudPAX process, to which all other methods tether.

This study concentrates primarily on the feasibility stage, Phase 1 (Discover) and Phase 2 (Define) (Figure 2). There is a period of overlap between these first two phases facilitating a cyclic-development between research and ideation. This allows continuous multidisciplinary review, and ensures an optimal balance is achieved within the design between functionality and user-friendliness. Comparatively, in Phase 2 an Alpha prototype might be generated; often in the overlapping stages between Phase 2 and 3. Later on in the final stages of the Phase 3 a Beta prototype will be generated.

The HudPAX research method is designed specifically to address the challenges associated with a collaborative, SME-centered, cross-sector medical design and development project, providing an optimized approach for academic and industrial collaboration. Tools including regular reviews, reports and quarterly board meetings help assess progress and ensure that the needs of all stakeholders are continually addressed.

### Evidence-Based Design Science Process

Design inputs and initial user requirements were gathered from previous pilot clinical studies, conducted by the Singapore team, to investigate the safety and feasibility of cryotherapy devices in preventing CIPN. These proof-of-concept studies investigated two cryotherapy modalities using existing class-I cooling devices designed for generic injury and therapeutic purposes. The cryotherapy modalities tested were continuous-flow cooling (Blanketrol III, Cincinnati SubZero, MA, USA), and cryocompression (Game Ready 2.0 Refurbished System, CoolSystems, Inc., CA, USA). Pilot studies involved healthy volunteers first, to investigate safety, tolerability and optimal parameters, and subsequently studied in cancer patients undergoing chemotherapy for efficacy in preventing CIPN (17–19). These studies involved subjects who underwent 3 h of limb cryotherapy (mimicking the duration of chemotherapy) of the upper limbs (fingers up to the elbow) and lower limbs (toes up to the knee). Next, cancer patients underwent up to 12 cycles of cryotherapy concomitantly with their chemotherapy. Overall, in these pilot studies, 56 healthy volunteers participated, and 33 cancer patients underwent a total of 360 cycles of cryotherapy concomitantly with their chemotherapy.

Design inputs were gathered from various stakeholders including the healthy volunteers and patients who participated in the study and, nurses and bioengineers who administered the cryotherapy. Design inputs gathered through the Design Science/evidence-based approach included scientific and clinical data which set the parameters for prototyping. Observations were made on the safety (discomfort or cold burns), temperature tolerability (intolerance to cooling and compression), therapeutic efficacy (co-administration with chemotherapy, coverage of wraps, and delivery of coolant) and comfort (fit and ease of use over extended duration of usage). These observations were made for both intra-cycle (set up, 3-h cryotherapy and suitability during any breaks/interruptions during cryo/chemotherapy) and over multiple cycles of chemotherapy.

### Design Considerations for Regulatory Compliance

Global medical device regulations are very similar and invoke a patient-protective, risk-based system where each device is classified into 1 of 4 categories. The higher the perceived risk, the more stringently the regulations are applied up to the highest risk where a full design dossier review is required.

With the exception of the Regulatory Quality management system (ISO 13485:2016), which forms the core of the HudPAX method, integration of regulatory standards is strategically limited during early-stage feasibility investigations of medical product design, in order to permit the full exploration of ideas to ensure the eventual selection of the optimal design. In accordance with the management system, regulatory standards should be fully addressed and applied in the later development stages (Phases 2, 3, and 4 in Figure 2), after feasibility studies are complete. In the subsequent stages of development, regulatory compliance will become a dominating factor for consideration and will significantly influence the device design. The complex challenge of attaining medical regulatory approval is a hurdle which medical device developments commonly fail to attain. In this project, integration of the commercial partner with extensive medical regulatory expertise mitigates this challenge.

While integration of regulatory standards is not required at this feasibility stage of the investigation, awareness of relevant impending regulations is important to ensure a smooth transition to the next development stage and to optimally position the development for eventual regulatory compliance. Regulatory standards which have been considered during this feasibility study include:

- IEC 10993 Biocompatibility is crucial to ensure that there are no adverse effects including irritation, infection and sores, as the limb wrap will be in direct contact with the users' skin. Due to the use of the Alpha prototype in focus group settings, due consideration of this standard was required in the feasibility stage.
- IEC 62366 stipulates usage, cleaning and movement of the device.
- IEC 62304 considers software applications, cybersecurity.
- IEC 60601-1-2 considers electromagnetic interference, both outbound and inbound.
- IEC 60601-1-11 addresses home use in all aspects, which is relevant since the commercial partner offers healthcare at home
- Safety and usability testing is considered in all aspects including, but not limited to:
  - Computer-Aided Design (CAD) where IEC 60601-1 was assessed for general requirements for basic safety and essential performance;
  - IEC 14971 was assessed for applications of risk management to medical devices;
  - IEC 62366 was assessed for applications of usability engineering to medical devices.

## Results - Design and Development

This study documents the design & development of an arm cryocompression wrap from a concept to a trial-ready prototype. The mixed-methods HudPAX approach promoted the strategic implementation of multiple complementary research methods, through the process of input gathering, ideation and prototyping and designing for manufacture, ultimately producing an Alpha prototype and facilitating a user-centered focus group study.

### Evidence Based Design Inputs

Design inputs were generated based on clinical evidence, which was gathered from over 400 cycles of cryotherapy using the two modalities: continuous-flow cooling, and cryocompression (17–19). These inputs formed the primary framework for generating feasibility considerations in the initial ideation phase. Inputs gathered include aspects pertinent to three key design requirement categories including: i. Compatibility with chemotherapy, ii. Safety and tolerability, and iii. Use over multiple cycles (Table 1).

**Table 1:** Design Requirements: Evidence-based design inputs.

**Compatibility with chemotherapy**	
Intravenous cannula insertion site	An important ergonomic consideration was the site of intravenous cannula insertion for chemotherapy on the arm, with respect to the wrap. During the treatment, it was a decisive design input for the nurses to be able to monitor the cannula site for signs of extravasation throughout the chemotherapy infusion. Moreover, during the scenario that the patient has an adverse reaction to the drugs, it is crucial that the cryotherapy arm wraps can be rapidly and easily detached to allow for safe resuscitation. Based on the pilot clinical studies in Singapore, the preferred cannula sites were on the dorsal (posterior) surface of the hands and palmar (anterior) of the forearm.
Breaks during chemotherapy	During chemotherapy, the patients tended to take up to three turns of toilet breaks. For sanitary purposes, the limb cooling wraps would have to be taken off during these breaks and fastened again. From our pilot studies, we observed that skin temperature changes during the toilet breaks were negligible and did not diminish the hypothermia effect. The wraps should be easy to unwrap and rewrap during these breaks.
Efficacious cryotherapy	Cryotherapy is hypothesized to offer optimal preventive effects for CIPN when sufficient cooling is achieved throughout the limbs. The wraps covering the arms from the fingertips up to the elbow would ensure maximal cooling of the periphery to enhance therapeutic efficacy.
**Safety and tolerability**	
Fit	The limb wraps should cater to fit various limb sizes across different ages, genders & ethnicities.
Comfort during extended durations of use	The wraps were fastened for an extended duration of 3 h during the pilot clinical studies. Sufficient space for free movement of finger and toe tips must be available. The wraps should also be designed in a manner to fit appropriately over the finger and toe tips, offer sufficient contact cooling and also ensure that too much pressure is not exerted causing pain and discomfort.
Tolerance to cryotherapy	Cryotherapy was generally well-tolerated by both healthy volunteers and cancer patients, in the pilot studies. Reports of intolerances were in the hands (fingertips) earlier than any intolerance in the rest of the arm. Coolant circulation throughout the wrap in an optimal pattern would enable stable coolant flow and considerable contact throughout the skin surface of the cooled extremities, the compression would further enhance the contact on the skin surfaces and improve tolerability.
**Use over multiple cycles**	
Hygiene and Storage	Cancer patients would undergo up to 12 cycles of chemotherapy and several patients were enrolled in the pilot studies. The cooling devices used in the study were disinfected and air dried before each use. Single-use/disposable or reusable with a removable outer sleeve/jacket would be suitable for use across a number of cycles of chemotherapy and in several patients. It is important that the material of the removable outer sleeve/jacket permits standard disinfection before and after every use.

### Ideation and Prototyping

**Figure 3:**
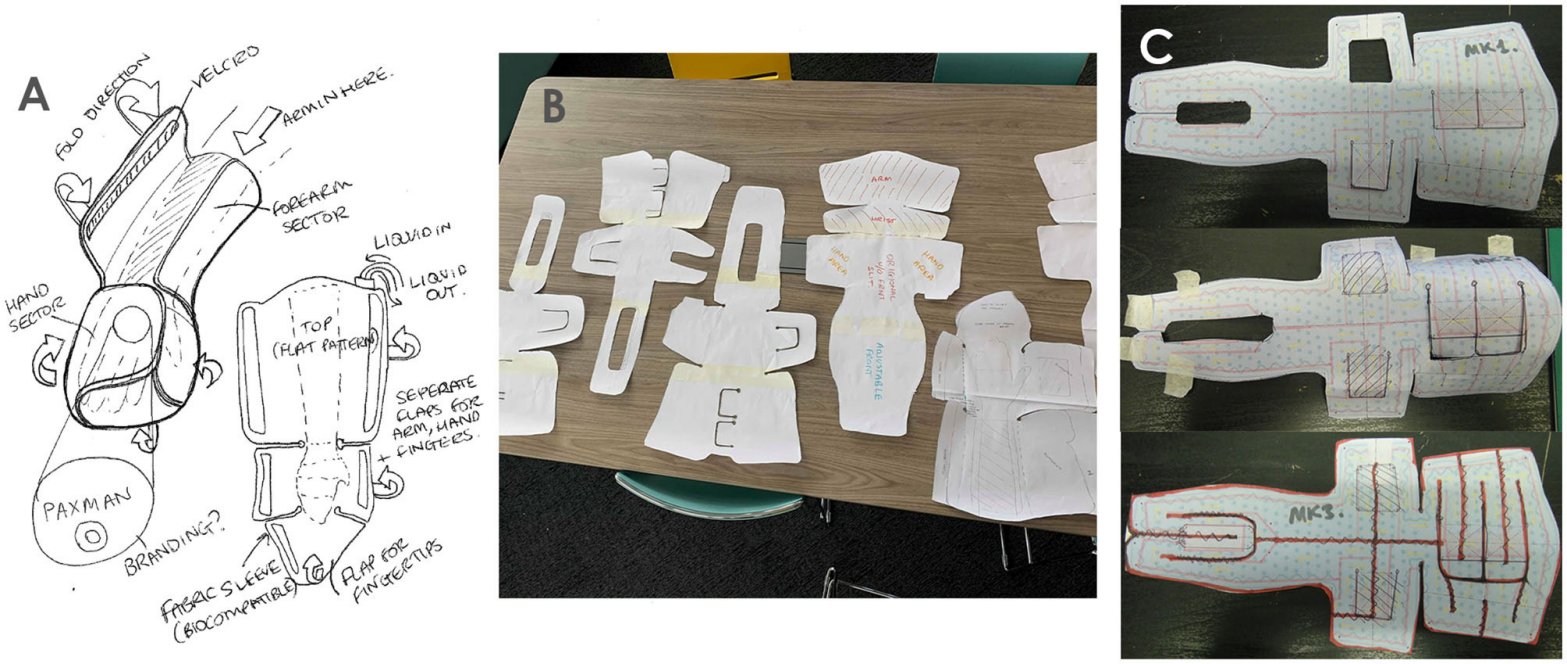
Ideation tools: Sketches **(A)** Paper sketch modeling - Form **(B)**, sketch modeling (technical considerations) **(C)**.

The HudPAX method integrates a well-established commercial product design & development framework, the Double Diamond design process, an incremental ideation approach to explore the full range of potential concepts. With the evidence-based design inputs as a foundation, Phase 1 (Discover - Research) and Phase 2 (Define - Ideate) were explored (Figure 2), primarily, through sketching, appearance modeling and sketch modeling techniques, utilizing low-cost materials including paper, card, and foam (Figure 3). These sketch models utilize fast and effective methods for evaluating concepts prior to investment into conventional or more expensive methods. The quantity of design concepts generated was open-ended, facilitating the consideration of a wide scope of ideas, and was subjective to finding the optimal design solution. The sketches were later translated into CAD models to enable detailing, including design for manufacturing elements and exploring the integration of user-centered aspects; the CAD data also made possible 3D printing.

An initial design concept was selected by the team, where ergonomic aspects were explored. The ergonomic foundation of this design was adjustability which allows the wraps to fit a wide variety of users' arm types/sizes, tightening it to different contours, and loosening if patients experience discomfort or sensitivity to the cold, in addition it provides easy removal for toilet breaks or in emergencies. The arm wrap will cover a large portion of the arm, hence many variables were to be addressed including: finger, palm and wrist size, forearm girth and arm length. Design mechanisms were used to allow for the required levels of adjustability including Velcro® and flaps for extending or reducing individual zones of the arm wrap. This development phase also involved the analysis of: suitable materials, exploration of potential manufacturing methods, testing, technical requirements (including flow rates and cooling efficiency), safety, regulatory standards, comfort, and aesthetics.

**Figure 4:**
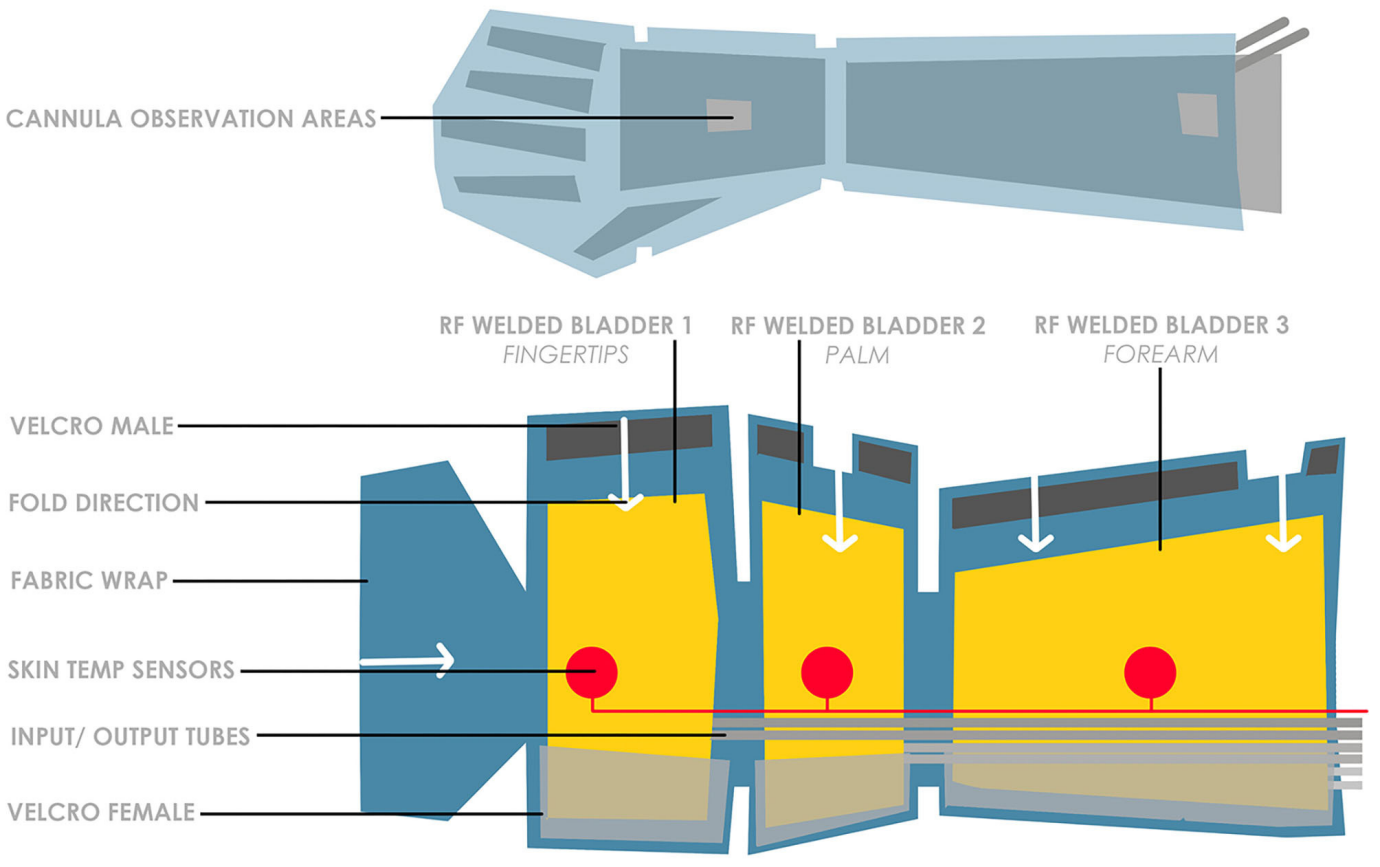
Three-bladder cooling zones arm wrap concept.

**Figure 5:**
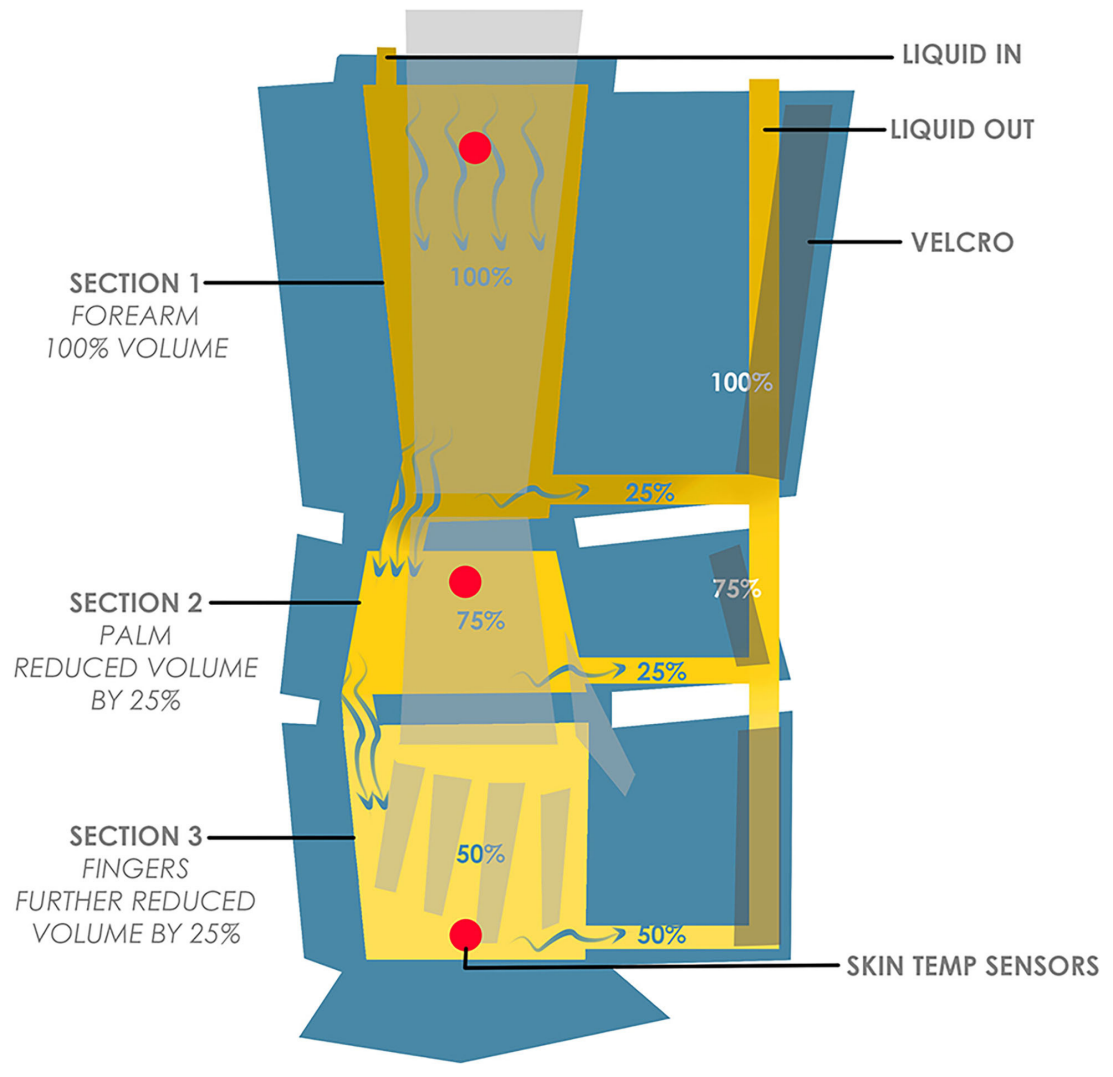
Bottleneck temperature regulation arm wrap concept.

The ergonomic design concept was implemented in the initial designs (Figures 4, 5) where considerations of form and function were integrated based on the evidence-based inputs including attachment method, intravenous cannula site access, cooling areas and ease of access.

Apart from evidence-based ergonomic inputs, the initial designs also included considerations of the optimal therapeutic parameters (best tolerable temperature and pressure ranges), based on pilot clinical trial efficacy data (17–19). Features such as integration of skin temperature sensors were explored to facilitate continual monitoring and feedback control, potentially enhancing treatment efficacy and patient comfort.

Based on the knowledge, from our pilot studies, that the forearm is able to tolerate lower temperatures than the fingertips, a feature of thermoregulating different zones was investigated through a concept which utilized three separate cooling bladders per arm (Figure 4).

Further exploration led to a concept with a single bladder which utilized a bottleneck design to reduce flow and adjust temperature incrementally from low, medium and high (with 50, 75, and 100% flow, respectively), through zones based on our clinical data on temperature tolerability (Figure 5). This single bottleneck bladder concept presented significant advantages over the multiple bladder concept, by minimizing the number of connectors, adaptors and tubing required, and limiting the weight of the device. In addition, it removes the issues associated with additional tubing which could tangle/kink and reduces the complexity of the refrigeration system or amount of systems required.

**Figure 6:**
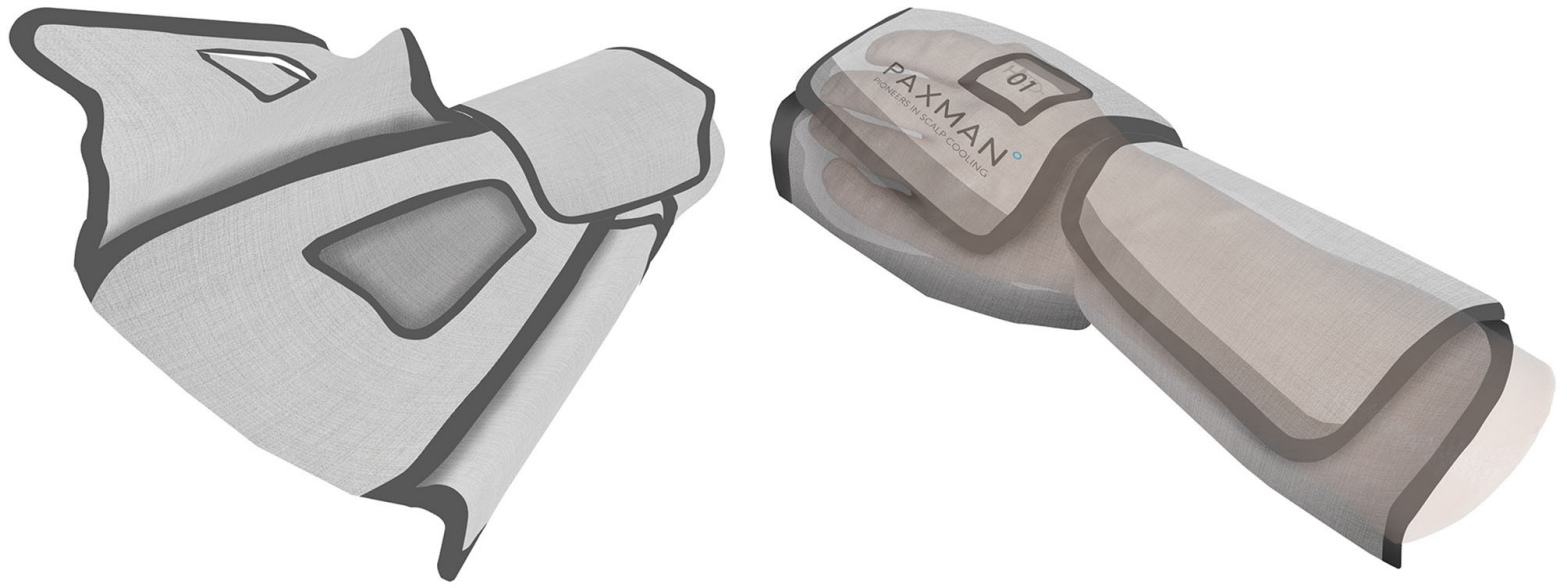
Initial chosen concepts for pre-clinical investigation.

**Figure 7:**
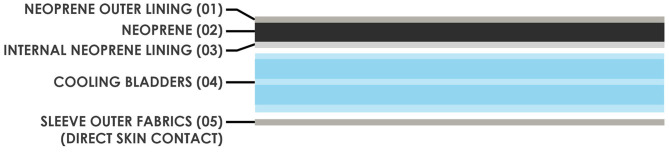
Cross-sectional analysis of the limb wrap materials and components.

The initial concept was then selected for pre-clinical investigation (Figure 6). Prototypes were created from the schematics, using a variety of medical grade fabrics considering crucial parameters such as biocompatibility and insulating properties (Figure 7). As this is wearable, sewing was used to create the outer shell of the wrap, which will encase and insulate the coolant bladder (Figure 7), to simulate usability, comfort, fit and access for intravenous cannula site. This provides a basis for generating a flat pattern in CAD which can be used as a Data Exchange File (DXF) by the manufacturing companies to prototype a Beta prototype.

**Figure 8:**
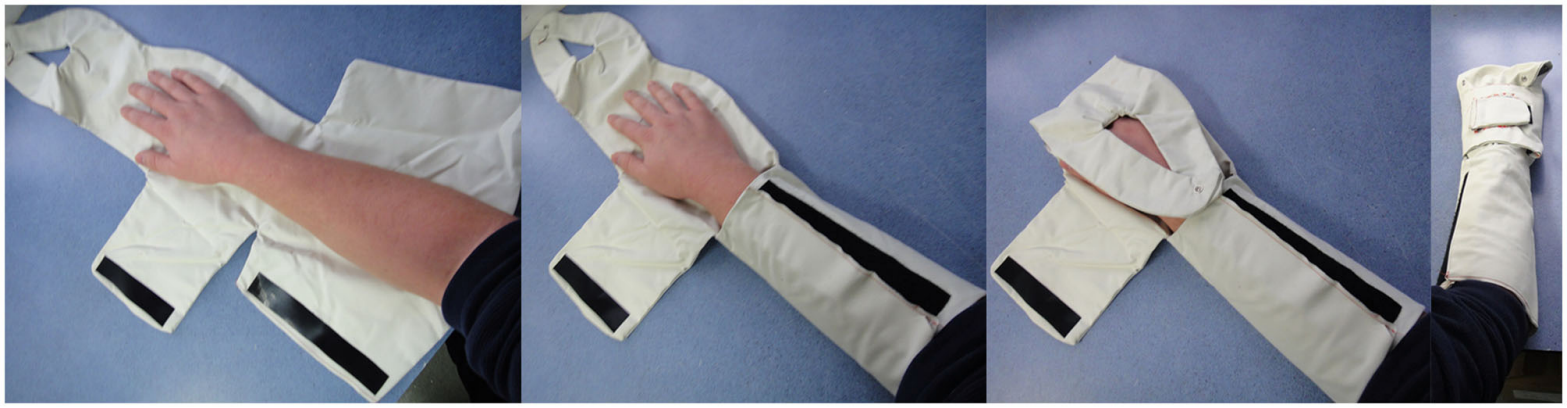
Fabric “Sketch Modeling” prototypes for usability testing.

**Figure 9:**
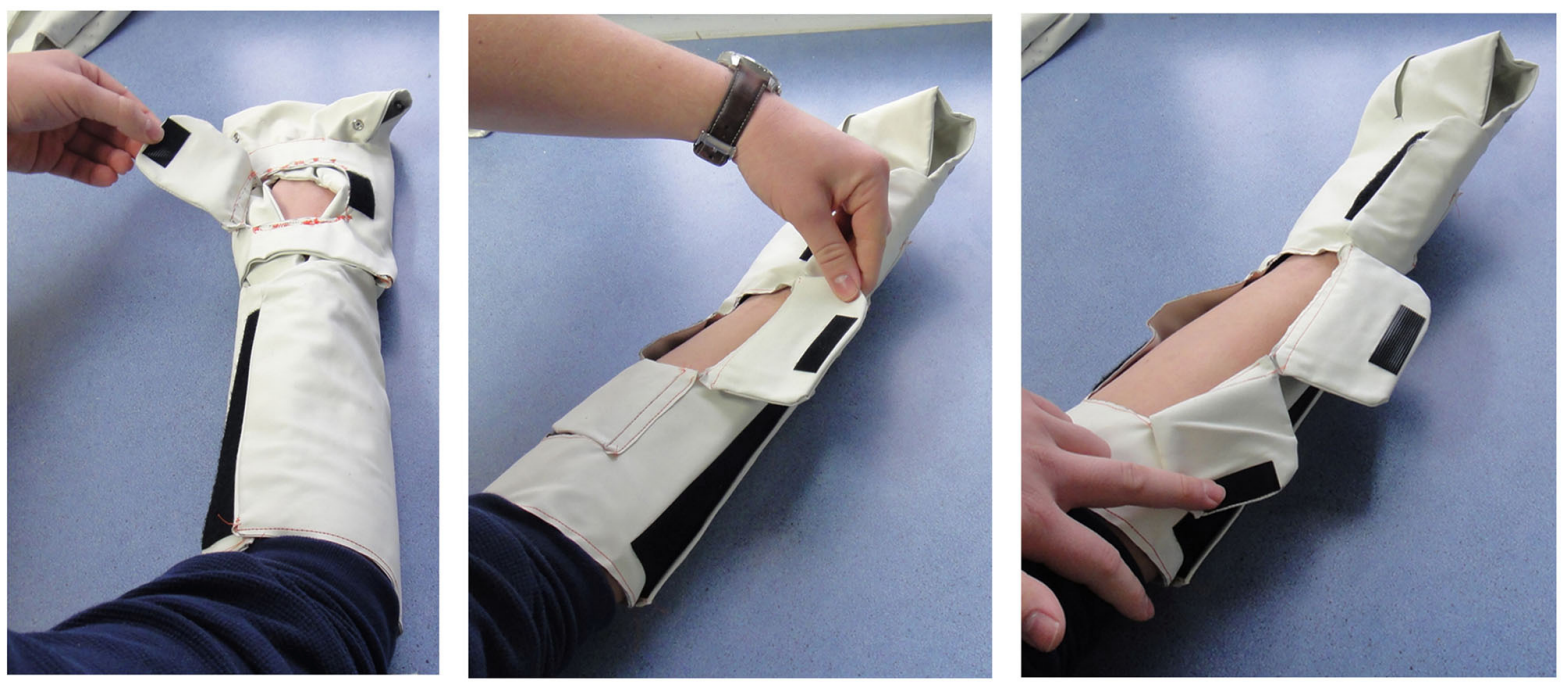
Intravenous cannula site windows in the “Sketch Modeling” phase.

The prototypes were created for a focus group testing with users and stakeholders (Figures 8, 9).

### User-Centered Stakeholder Focus Group Study

The developed Alpha prototypes were presented to a stakeholder focus group to gather user-centered and ergonomic based feedback. The user-centered focus group study was conducted at the Clinical Research Facility, Leeds teaching hospital, Leeds, UK and involved healthcare professionals and patient and public involvement (PPI) groups. Engagement of PPI groups ensures that patients, public and caregivers are actively involved early on in the medical device design process and their insights and perspectives are taken into account. This user perspective also aims to mitigate any possible oversights at an early stage in the medical device development process.

The focus group, consisting of 12 healthcare professionals and PPI group members, acknowledged the unmet clinical need and appreciated the concept of a wearable device to prevent CIPN. Participants were informed on the usability aspects of the wrap design including compression and cooling aspects, correct fit, intravenous cannula monitoring, ease of removal, optional pressure on the cannulated arm and instructions for use. Participants were encouraged to try on the prototype wraps alongside examples of existing non-CIPN specific cooling devices. The following user-centered development aspects were explored:

#### Intravenous Cannula Site Window

**Figure 10:**
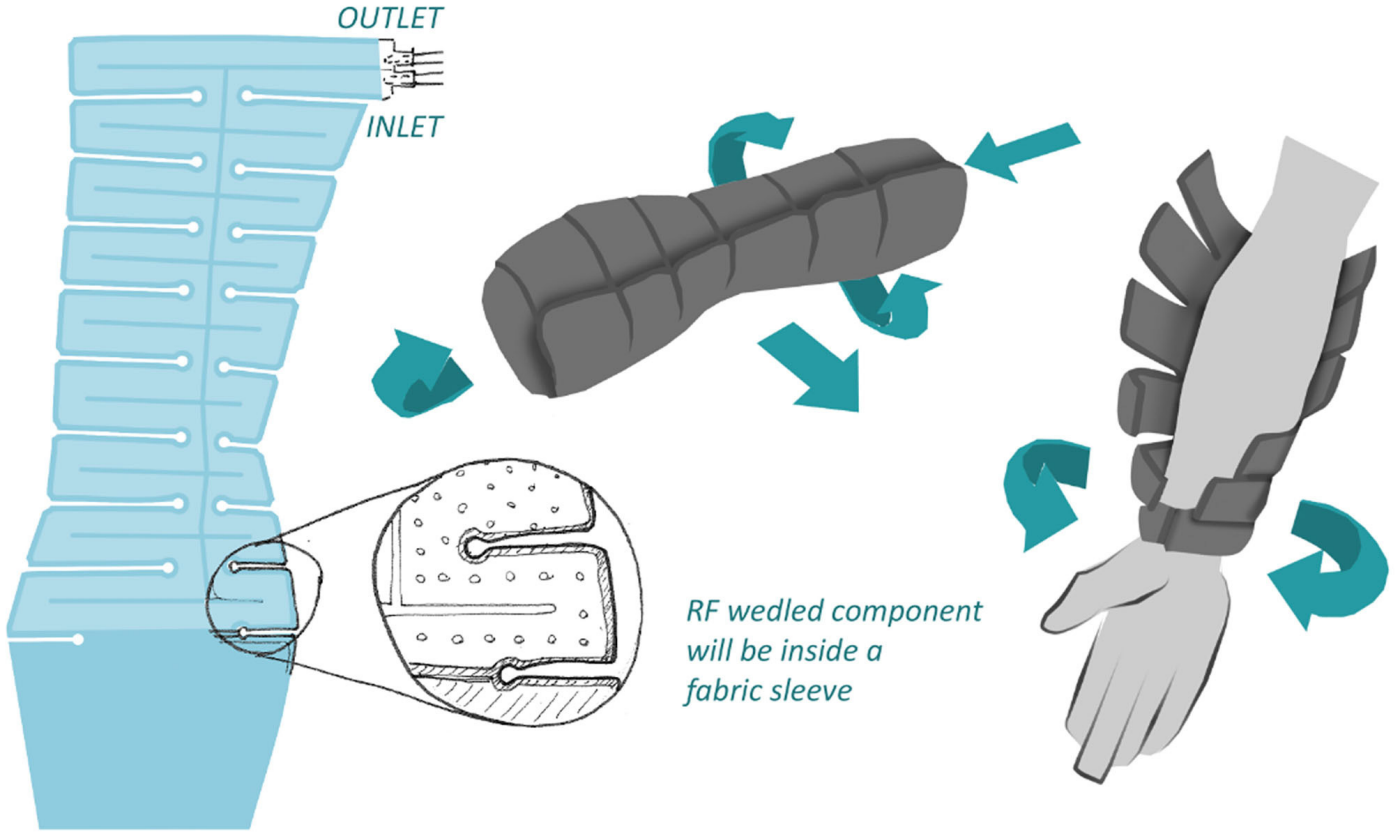
Limb cooling wrap concept developed based on the Focus group feedback.

The focus group appreciated the idea of an intravenous cannula site window providing visual access to the healthcare professionals to monitor any extravasation or adverse reactions that might occur during the chemotherapy infusion. At the same time, this allows for the cannulated arm to receive the benefit of cryotherapy. The placement and size of the intravenous cannula site window in the Alpha prototype was suggested to be modified to fit cannulation practices in the UK, as the earlier placement was catered to practice in Singapore, based on the pilot clinical trial evidence. This highlighted a key user-centered design aspect to be taken into consideration. Using these key inputs, we applied the data from the focus group to develop a concept prototype including multiple adjustable flaps throughout the wrap, as shown in Figure 10, which could be adapted to facilitate universal cannulation practices.

#### Usability During Chemotherapy

The focus group suggested incorporation of a design feature for the intravenous feed to fit through the wrap without dislodging the cannula. This would allow for the healthcare professionals to remove the wrap without removing the cannula to facilitate toilet breaks.

#### Reusability of Wraps

The focus group considered the reusability of wraps and a suitable biocompatible material which would allow for disinfection and reuse over different chemotherapy cycles and in different patients.

#### Comfort and Fit

The Velcro® flaps were reported to be simple, easy and convenient to use, especially for quick wrap and unwrap during toilet breaks. The focus group was comfortable with the fit of the prototype wraps which catered to various sizes of arms and ease of adjustability. They also indicated that there was sufficient “breathing” space around the palm area to allow fingers movement during the extended period of application. A concern was that the wrap may be too long for smaller sizes of arms. The group also considered the weight of the device exerted on the patient's limbs as a factor of comfort through the entire duration of cryotherapy.

The next stages of prototype development will involve the production of functional or proof-of-concept prototypes which allow the retention and flow of liquids and air pressure, thus allowing the evaluation of fluid dynamics, temperature control, pressure and temperature tolerability. The feedback gathered in this study was adopted as design inputs into the design development process. This further led to the developed Figure 10 which includes improvements and modifications of the Alpha prototype design which was tested in the user-centered focus group study (Figures 8, 9).

### Design for Manufacture

Following on from this feasibility study, in phase 2 and phase 3 of the methodology, the design for manufacture becomes a more dominant consideration. In phase 2, we collaborate with several manufacturing companies with different specialist areas to make Alpha prototypes and low-volume prototyping for validation testing and clinical trials. Considerations include the materials, methods and the costs associated with one-off or low-volume manufacture. The later stages will increase the focus on the design for manufacture, specifically regarding tooling, where rapid tooling and hard tooling will be considered depending on volumes, quality and constraints for both the external limb-wrap and the internal coolant-containing-bladder. Due to the many parallels between this device and Paxman's established scalp cooling device, the existing industry expertise and infrastructure for prototyping, supply and material usage (i.e., biocompatibility) provides a readily-available framework which can be adopted and modified to the unique requirements of this project.

For the manufacture of the internal bladder for the functional prototype, Radio-Frequency (RF) welding has been selected. RF welding uses high frequency electromagnetic energy to generate heat, thus bonding sheets of plastic together (29). Technical parameters have been set to investigate developments for regulatory compliance. Crucial factors include sanitation, biocompatibility, strength, flexibility, ability to hold pressurized air and liquid. Material analysis for the internal bladder led to the selection of 300 μ TPU film, for its biocompatibility, flexibility, manufacturability, wear and tear resistance. To generate a low-volume prototype run using RF welding without the aid of tooling, CAD data is required. Using the CAD software SolidWorks, flat patterns of the physical model are converted into a DXF file, which can be utilized by industry to manufacture the sample for testing.

## Discussion

This study documents the design and development of arm wraps as a central component of a CIPN-prevention limb cryocompression system. Using the HudPAX method, design input requirements were gathered from existing clinical pilot studies, and design outputs were developed in an iterative process, with user-centered approaches providing vital stakeholder feedback and propelling the development into its next stage. This research demonstrates the importance of cross-sector multidisciplinary collaboration, mixed-methodology adaptation and the integration of stakeholder consultation, in addressing these challenges, particularly with SME-centered developments where resources and research and development infrastructures are limited.

### Next Steps in Device Development

Our ultimate aim is to generate a complete four-limb cryocompression system for CIPN prevention, which can be utilized by patients worldwide, alongside the existing Paxman scalp cooling system. The roadmap of milestones to ensure the continuation of this successful development includes:

- Development of a Beta prototype - based on the findings of this study, generating a near-final design for use in the subsequent clinical trials;
- A healthy volunteer trial to determine safety will be conducted using the Beta prototype;
- A clinical trial in cancer patients undergoing chemotherapy will be conducted, to prove efficacy;
- Regulatory approval will be obtained;
- The “detailing and final prototype” stage in collaboration with industry will finalize the product;
- The final product will be launched to an international market, through Paxman's existing infrastructure.

### Challenges in Multidisciplinary Medical Product Development

The method adopted in this project was developed to address the complex and high-risk nature of SME-centered medical device design & development projects and supports the equilibrium between the distinct needs of medical device design, user needs, commercial feasibility and stringent regulatory standards. The complex challenges encountered within medical device development demands a multidisciplinary approach; in which experts from all fields of relevant research collaborate to address unmet clinical needs. In this project, early-stage clinical trials generated the evidence-based inputs and ensured the feasibility of the potential solution. The subsequent ideation phase engaged the expertise of designers and engineers, who utilized the scientific evidence to generate potential concepts. Qualitative assessment of the concepts, from users, was then essential to progress the development into the next stages, where opinions and motivations are explored. This fundamental communication between Design Science and Design Thinking methods is the foundation for establishing early success in medical device design & development.

#### Industrial Commercial Awareness

As we have explored in this paper, medical device design & development is complex. The development process needs to be adaptable and multifaceted to ensure the commercial viability. A crucial consideration for any development, during the discovery phase (Phase 1 - Research), is a market analysis to assess the market for existing products, state-of-the-art technology and industrial feasibility. A comprehensive literature review identified key commercial aspects including market size, freedom to operate, problem definition, user behavior, opportunities and needs, together with the integration of academic aspects.

#### Standards Regulations

Ultimately this development aims to achieve MDSAP (Medical Device Single Audit Program) approval, which is medical device compliance encompassing HC, FDA, ANVISA, EC, ATG and PMDA, and represents six different medical device markets: Australia, Brazil, Canada, Europe, Japan and the United States. Regulatory approval for additional markets will also be strategically pursued. Utilizing the existing knowledge and infrastructure available from the commercial partner, the future challenges associated with obtaining regulatory compliance, which commonly account for new medical devices' market failure, will be mitigated.

#### Market Considerations and Scalp-Cooling Parallels

Approximately 30,000 patients in the UK, and 1 million worldwide, can be affected by CIPN annually. Scalp-cooling prevents CIA, which occurs alongside CIPN and therefore the market already exists. Collaboration with Paxman Coolers Ltd integrates into the project an existing route-to-market with the synergies facilitating improved productivity and economies-of-scale to be achieved. Limitations specific to this CIPN prevention device include the additional medical time required to attach/detach/monitor the therapy; the additional space required for the device within hospital/clinics; and also potential low adoption of the therapy due to current low-level awareness amongst patients of CIPN as a side-effect of chemotherapy. Identification and consideration of potential limitations by the multidisciplinary team and stakeholders, at this feasibility stage, facilitates early mitigation.

## Conclusion

This paper has addressed the challenges posed by early-stage medical device design & development, including the following challenge areas: *Medical requirements; Technical requirements; Stakeholder and user requirements; Regulatory requirements; Multidisciplinary team varied expectations; Commercial viability; and Market awareness and accessibility*.

Through the development of a limb-hypothermia arm wrap Alpha prototype for CIPN prevention, cross-sector collaboration has united multidisciplinary expertise from clinical, academic and commercial backgrounds, with the combined regulatory and market-awareness knowledge providing a strong foundation which proves central to the development's ultimate viability.

Development and implementation of a mixed-methodology has promoted a flexible approach addressing the complex design challenges and optimized to the cross-sector needs and expectations of the collaborating partners. This mixed-methodology has also demonstrated the equal importance of evidence-based and user-centered inputs, primarily for setting the clinical and technical parameters and secondly for integrating user requirements. Our work highlights the necessity for stakeholder consultation in the early stages of medical device development, specifically when multiple user-types (nurses/patients) exist, to ensure user-inputs comprehensively reflect the user-needs. Continuation of the mixed-methods approach will propel the device through the next stages of the development including Beta prototyping, clinical trials and eventual market launch.

## Data Availability Statement

The original contributions generated for the study are included in the article/supplementary material, further inquiries can be directed to the corresponding author/s.

## Ethics Statement

The studies involving human participants were reviewed and approved by School Research Ethics and Integrity Committee (SREIC), University of Huddersfield, UK. The patients/participants provided their written informed consent to participate in this study. Written informed consent was obtained from the individual(s) for the publication of any potentially identifiable images or data included in this article.

## Author Contributions

JB, EU, PB, RP, RS, and AB contributed to the conception and design of research. JB and AB contributed to the implementation of the research, acquisition, and interpretation of data. JB, EU, JC, and AB drafted the manuscript. PB, RP, and RS revised the manuscript critically and for important intellectual content. All authors read and approved the final manuscript.

## Conflict of Interest

JB and PB are employed by Paxman Coolers Ltd. RP partially owns Paxman Coolers Ltd. EU, RS, and AB have received research or travel funding from Paxman Coolers Ltd. The remaining author declares that the research was conducted in the absence of any commercial or financial relationships that could be construed as a potential conflict of interest. This work was supported by Paxman Coolers Ltd., UK. The funder was involved in the commercial design decisions as this project is a commercial product design and development (not medical study), but the academic and research team independently made decisions for data collection, analysis, interpretation of data, the writing of this article and the decision to submit it for publication.

## Abbreviations

ANVISA: Agência Nacional de Vigilância Sanitária/National Sanitary Surveillance Agency
ATG: Australian Therapeutic Goods Administration
CAD: Computer Aided Design
CIA: Chemotherapy Induced Alopecia
CIPN: Chemotherapy-induced peripheral neuropathy
DXF: Data Exchange File
EC: European Commission
FDA: Food and Drug Administration
HC: Health Canada
IEC: International Electrotechnical Commission
ISO: International Organization for Standardization
MDSAP: Medical Device Single Audit Program
PMDA: Pharmaceuticals and Medical Devices Agency
PPI: Patient and Public Involvement
RF: Radio Frequency
SME: Small to Medium Enterprise.
